# Phase 1 study of the pan-RAF inhibitor tovorafenib in patients with advanced solid tumors followed by dose expansion in patients with metastatic melanoma

**DOI:** 10.1007/s00280-023-04544-5

**Published:** 2023-05-23

**Authors:** Drew W. Rasco, Theresa Medina, Pippa Corrie, Anna C. Pavlick, Mark R. Middleton, Paul Lorigan, Chris Hebert, Ruth Plummer, James Larkin, Sanjiv S. Agarwala, Adil I. Daud, Jiaheng Qiu, Viviana Bozon, Michelle Kneissl, Elly Barry, Anthony J. Olszanski

**Affiliations:** 1 South Texas Accelerated Research Therapeutics, LLC, San Antonio, TX USA; 2 University of Colorado Cancer Center, Aurora, CO USA; 3 Department of Oncology, Cambridge University Hospitals NHS Foundation Trust, Cambridge, UK; 4 Laura & Isaac Perlmutter Cancer Center at NYU Langone, New York, NY USA; 5 Department of Oncology, NIHR Biomedical Research Centre, Oxford, UK; 6 The Christie NHS Foundation Trust and Division of Cancer Sciences, University of Manchester, Manchester, UK; 7 Bristol Haematology and Oncology Centre, Bristol, UK; 8 The Northern Centre for Cancer Care, Freeman Hospital, Newcastle upon Tyne, UK; 9 The Royal Marsden Hospital, London, UK; 10 St. Luke’s Cancer Center and Temple University, Easton, PA USA; 11 UCSF Helen Diller Family Comprehensive Cancer Center, San Francisco, CA USA; 12 Day One Biopharmaceuticals, 2000 Sierra Point Parkway, Suite 501, Brisbane, CA 94005 USA; 13 Millennium Pharmaceuticals, Inc., a wholly owned subsidiary of Takeda Pharmaceutical Company Limited, Cambridge, MA USA; 14 Fox Chase Cancer Center, Philadelphia, PA USA

**Keywords:** Tovorafenib, Pan-RAF inhibitor, BRAF, Phase 1, Melanoma

## Abstract

**Purpose:**

Genomic alterations of *BRAF* and *NRAS* are oncogenic drivers in malignant melanoma and other solid tumors. Tovorafenib is an investigational, oral, selective, CNS-penetrant, small molecule, type II pan‑RAF inhibitor. This first-in-human phase 1 study explored the safety and antitumor activity of tovorafenib.

**Methods:**

This two-part study in adult patients with relapsed or refractory advanced solid tumors included a dose escalation phase and a dose expansion phase including molecularly defined cohorts of patients with melanoma. Primary objectives were to evaluate the safety of tovorafenib administered once every other day (Q2D) or once weekly (QW), and to determine the maximum-tolerated and recommended phase 2 dose (RP2D) on these schedules. Secondary objectives included evaluation of antitumor activity and tovorafenib pharmacokinetics.

**Results:**

Tovorafenib was administered to 149 patients (Q2D *n* = 110, QW *n* = 39). The RP2D of tovorafenib was defined as 200 mg Q2D or 600 mg QW. In the dose expansion phase, 58 (73%) of 80 patients in Q2D cohorts and 9 (47%) of 19 in the QW cohort had grade ≥ 3 adverse events. The most common of these overall were anemia (14 patients, 14%) and maculo-papular rash (8 patients, 8%). Responses were seen in 10 (15%) of 68 evaluable patients in the Q2D expansion phase, including in 8 of 16 (50%) patients with *BRAF* mutation-positive melanoma naïve to RAF and MEK inhibitors. In the QW dose expansion phase, there were no responses in 17 evaluable patients with *NRAS* mutation-positive melanoma naïve to RAF and MEK inhibitors; 9 patients (53%) had a best response of stable disease. QW dose administration was associated with minimal accumulation of tovorafenib in systemic circulation in the dose range of 400–800 mg.

**Conclusions:**

The safety profile of both schedules was acceptable, with QW dosing at the RP2D of 600 mg QW preferred for future clinical studies. Antitumor activity of tovorafenib in *BRAF*-mutated melanoma was promising and justifies continued clinical development across multiple settings.

**ClinicalTrials.gov identifier:**

NCT01425008.

**Supplementary Information:**

The online version contains supplementary material available at 10.1007/s00280-023-04544-5.

## Introduction

The mitogen-activated protein kinase/extracellular signal regulated kinase (MAPK/ERK) pathway, comprising RAS, RAF, MEK and ERK proteins, couples extracellular growth factor signals from receptor tyrosine kinases to intracellular responses, modulating cell proliferation and survival [1, 2]. Mutations in components of this pathway have been shown to be oncogenic drivers in a wide range of human cancers. Activating point mutations of the *BRAF* gene encoding the serine/threonine-protein kinase BRAF have been identified in 50–60% of malignant melanomas, and at similar or lower frequencies in several other cancers [3–6]. In melanoma, many of the tumors which are *BRAF* wild-type carry activating mutations of *NRAS*, which encodes another component of the MAPK pathway positioned above RAF in the signaling cascade [5].

In around 70–90% of *BRAF-*driven tumors, the activating genomic alteration is a mutation resulting in the substitution of valine (V) for glutamic acid (E) in the BRAF kinase domain (V600E) [6]. The V600E mutation allows BRAF to adopt a constitutively active conformation in the absence of dimerization, permitting RAS-independent monomer signaling [7]. Other *BRAF* alterations, including *BRAF* gene fusions seen in the majority of pediatric pilocytic astrocytomas, encode constitutively active RAS-independent dimers [8–10]. Initial efforts to therapeutically target BRAF focused on the development of small molecule type I inhibitors that showed high specificity towards the V600E mutant. This led to the regulatory approval of vemurafenib, dabrafenib and encorafenib (and combinations with MEK inhibitors and other agents) for the treatment of patients with unresectable or metastatic melanoma harboring a BRAF V600E mutation [11–16]. In relation to the optimal sequencing of treatment in this setting, the phase 3 DREAMseq trial showed that the combination of nivolumab/ipilimumab followed by BRAF/MEK inhibitor therapy, if necessary, should be the preferred sequence for patients with *BRAF*-mutated advanced melanoma [17].

Type I inhibitors are not indicated for the treatment of patients with BRAF wild-type melanoma or melanomas harboring oncogenic *BRAF* fusions. In BRAF wild-type cells, type I BRAF inhibitors can paradoxically cause MAPK activation due to BRAF-inhibitor-mediated homodimerization and heterodimerization of nonmutant RAF isoforms. Type I inhibitor binding to one protomer of a wild-type RAF dimer causes allosteric transactivation of the other protomer, while at the same time reducing the affinity of the drug for that other protomer, resulting in enhanced signaling [18, 19]. This characteristic may underlie the development of cutaneous squamous-cell carcinomas and other secondary malignancies in patients treated with type I BRAF inhibitors, many of which harbor activating RAS mutations [20, 21].

Tovorafenib (also known as DAY101, TAK-580, MLN2480, BIIB024) is an investigational, oral, central nervous system-penetrant, selective, small molecule pan-RAF kinase type II inhibitor. It shows potent activity against BRAF V600E and oncogenic BRAF fusions, suppressing the activity of both monomeric and dimeric forms [9]. In preclinical studies, tovorafenib has been shown to be a potent pan-RAF inhibitor in biochemical kinase assays with IC_50_ values of 7.1, 10.1 and 0.7 nM for the BRAF V600E mutant, wild-type BRAF and wild-type CRAF, respectively. In addition, tovorafenib showed strong and sustained p-ERK suppression in pharmacodynamic studies with *BRAF*-mutant, *BRAF* deletion mutation and *NRAS*-mutant xenograft tumor models and caused tumor regression in large, established BRAF V600 mutant melanoma xenografts in mice, with tumors remaining sensitive to a second dosing cycle [22]. Further nonclinical studies in model systems showed that tovorafenib has good blood–brain barrier penetration of healthy brain as well as of intracranial tumors generated by stereotactic injection of pediatric low-grade astrocytoma cells harboring a *KIAA1549*-*BRAF* fusion. In addition, it was shown not to trigger paradoxical activation of ERK signaling in neural progenitor cells transformed with KIAA1549-BRAF fusion protein, and to bind with equal affinity to monomeric and dimeric forms of BRAF [9].

The aim of this phase 1 dose escalation study was to evaluate the safety profile and to determine the maximum tolerated dose (MTD) of tovorafenib given as monotherapy on once every other day (Q2D) and once weekly (QW) dosing regimens to patients with relapsed or refractory solid tumors. In a subsequent dose expansion phase of the study, the safety and preliminary antitumor activity of tovorafenib were further evaluated in cohorts of patients with *BRAF*-mutant, *NRAS*-mutant and *BRAF/NRAS* wild-type metastatic melanoma.

## Patients and methods

### Study design

We conducted a first-in-human, phase 1, multicenter, open-label study of tovorafenib in patients with relapsed or refractory advanced solid tumors. The study included a dose escalation phase and a dose expansion phase, with the latter including molecularly defined cohorts of patients with locally advanced, metastatic, and/or unresectable melanoma. The primary objectives were to evaluate the safety and tolerability of tovorafenib administered either Q2D or QW, and to determine the MTD and recommended phase 2 dose (RP2D) on these schedules. Secondary objectives were to evaluate the preliminary antitumor activity and pharmacokinetics (PK) of tovorafenib and to assess the effect of tovorafenib on pharmacodynamic markers in paired tumor biopsies.

The protocol was approved by the institutional review boards or independent ethics committees of all participating centers. The study was conducted in accordance with the protocol, Declaration of Helsinki, International Council on Harmonisation Good Clinical Practice guidelines, and applicable regulatory requirements. Written informed consent was obtained from all patients or their guardian/legal representative before study participation.

### Patients

Eligible patients were aged ≥ 18 years, with relapsed or refractory advanced solid tumors (excluding lymphoma but including melanoma), who had progressed on/after, or were not candidates for standard therapies or for whom no approved therapy was available (dose escalation phase and PK cohort of dose expansion phase) or who had locally advanced, metastatic, and/or unresectable melanoma which met predefined cohort-specific molecular or prior treatment criteria (dose expansion phase; Supplementary Table S1). Other key eligibility requirements included an Eastern Cooperative Oncology Group performance status ≤ 1, an expected survival time of at least 3 months, thyroid function tests consistent with stable thyroid function, a left ventricular ejection fraction of 50% or greater, as measured by echocardiogram or multiple-gated acquisition scan performed within 28 days before the first dose of tovorafenib, and suitable venous access for study-required blood sampling.

Previous chemotherapy and hormone therapy were to have been completed at least 4 weeks or 4 half-lives, whichever occurred first, prior to administration of tovorafenib. Previous immunotherapy/monoclonal antibody use had to have been completed at least 4 weeks and radiation therapy at least 3 weeks prior to the administration of tovorafenib. Prior treatment with programmed cell death protein 1 (PD-1) and programmed cell death 1 ligand 1 (PD-L1) monoclonal antibodies was permitted with a washout period of ≥ 6 weeks, provided there was no observed tumor shrinkage during that time relative to the previous progression scan. All associated toxicity from previous therapies had to have been resolved to ≤ grade 1 prior to administration of tovorafenib.

Exclusion criteria included a history of any major disease that might interfere with safe protocol participation, inadequate organ function (absolute neutrophil count [ANC] ≤ 1500/μL; platelet count ≤ 75,000/μL; hemoglobin < 9 g/dL [hemoglobin could be supported by transfusion, erythropoietin, or other approved hematopoietic growth factors]; serum bilirubin ≥ 1.5 × upper limit of normal (ULN) or ≥ 2 × ULN if the patient was known to have Gilbert’s Disease as the only underlying hepatic disorder; aspartate aminotransferase (AST) and alanine aminotransferase (ALT) ≥ 2.5 × ULN (AST and ALT ≥ 5 × ULN for patients with liver metastasis); serum creatinine ≥ 2.0 mg/dL); brain metastasis, unless previously treated with surgery, whole-brain radiation, or stereotactic radiosurgery and the disease had been stable for at least 2 months without steroid use or on a stable dose of steroids for at least 1 month prior to the first dose of tovorafenib; other active malignancy (dose expansion phase); evidence of current uncontrolled cardiovascular conditions; active bacterial or viral infection; prior investigational agents for malignant or non-malignant disease or major surgery within 28 days, or treatment with strong or moderate CYP3A/CYP2C inducers or gemfibrozil (strong CYP2C8 inhibitor) within 14 days, before the first dose of study drug. Female patients were excluded if they were pregnant or breastfeeding.

### Procedures

Tovorafenib was administered orally (tablet formulation), with patients fasting (except for water) for at least 2 h before and at least 2 h after taking their dose. Treatment was to be continued until disease progression, unacceptable toxicity, or the patient discontinued for any other reason, for a maximum duration of 12 months. Treatment could be continued beyond 12 months if it was determined that a patient would derive benefit from such continued therapy.

In the dose escalation phase, a 3 + 3 design was used to evaluate tovorafenib administered with continuous dosing on Q2D and QW dosing regimens. Prior to the initiation of QW dose escalation, the initial cycle length of 22 days was changed to 28 days by protocol amendment to improve clinical feasibility and better facilitate future combination studies. Patients enrolled prior to this protocol amendment in an ongoing Q2D dose escalation cohort continued on the 22-day cycle schedule until the cohort was full and all patients had been evaluated for dose-limiting toxicity (DLT). For both Q2D and QW regimens, dose escalation progressed according to the incidence of DLT in the first treatment cycle (either 22 days or 28 days). DLTs were defined as: grade 4 neutropenia lasting ≥ 7 consecutive days; febrile neutropenia (defined as an ANC ≤ 1000 cells/μL and fever ≥ 38.5 °C) or documented infection ≥ grade 3 with ANC ≤ 1000 cells/μL; grade 4 thrombocytopenia (platelet count < 25,000/μL), tovorafenib-related thrombocytopenia requiring platelet transfusion, or tovorafenib-related bleeding requiring medical attention; treatment delays of ≥ 14 days due to any toxicity; ALT and AST toxicities (ALT or AST > 7.5 × ULN for greater than 14 days or ALT or AST > 7.5 × ULN accompanied by an elevation in total bilirubin of > 3 × ULN [not explained by obstruction] regardless of duration); nonhematological toxicity ≥ grade 3 (with the exception of: nausea, vomiting, and diarrhea except if they persisted at ≥ grade 3 for > 3 days despite adequate supportive care measures [at the investigator’s discretion, patients who experienced nausea, vomiting, or diarrhea after taking tovorafenib could receive antiemetic or antidiarrheal medication prior to subsequent doses]; isolated laboratory abnormalities ≥ grade 3 that resolved to ≤ grade 1 in ≤ 7 days without clinical sequelae or the need for therapeutic intervention; fatigue ≥ grade 3 for ≤ 7 days; development of keratoacanthomas or skin carcinoma unless unusually aggressive or metastatic), provided the site investigator considered such events were at least possibly related to study treatment. The MTD was defined as the highest dose level that generated DLT in 0/3 or 1/6 patients. On a case-by-case basis, the sponsor in collaboration with the principal investigators determined if intrapatient dose escalation was appropriate. Patients who had any dose reductions were not permitted to dose escalate.

The starting dose for the Q2D dose escalation phase was 20 mg, which was equivalent to one-tenth of the highest non-severely toxic dose (HNSTD) established in monkey toxicology studies. Dose escalation included planned dose levels of 40 mg, 80 mg, 135 mg, 200 mg, and 280 mg. Once the MTD and/or RP2D of Q2D tovorafenib was established, patients with melanoma were enrolled into 1 of 6 Q2D melanoma expansion cohorts (approximately 16 patients per cohort), based on tumor genotype and treatment history (Supplementary Table S1). In addition, a seventh Q2D cohort was to enroll sufficient patients (approximately 16) with any advanced solid tumor (excluding lymphoma) to ensure that 12 patients completed protocol-specified dosing and PK assessments scheduled during cycle 1.

The study was initially designed to investigate a Q2D schedule. Subsequently, a protocol amendment introduced planned QW dose escalation cohorts. The alteration in the dosing regimen from Q2D to QW was expected to reduce drug accumulation and increase *C*_max_ while maintaining similar steady-state AUC. In addition, it was hypothesized that the increased *C*_max_ might lead to a higher degree of pathway inhibition for a window of time within the dosing interval, without compromising overall dose density. Planned QW doses to be administered on days 1, 8, 15, and 22 of a 28-day cycle were a starting dose of 400 mg, followed by dose level increases of 200 mg (i.e., doses of 600 mg, 800 mg, and 1000 mg) in each subsequent cohort until the MTD/RP2D was reached. Once the MTD and/or RP2D of Q2D tovorafenib was established, and following a further protocol amendment, a single expansion cohort of up to 16 patients with *NRAS*-mutated cutaneous melanoma, naïve to prior therapy with RAF and MEK inhibitors was enrolled.

### Safety, pharmacokinetic and pharmacodynamic assessments

Adverse events were coded using the Medical Dictionary for Regulatory Activities (MedDRA) Version 19.0 and were graded according to the National Cancer Institute (NCI) Common Terminology Criteria (CTC) for adverse events (CTCAE) (Version 4.03). The assessment period for treatment emergent adverse events (TEAEs) was from the first dose of study treatment to 30 days after the last dose of study medication, or until the start of subsequent antineoplastic therapy, whichever occurred first. Following baseline evaluation, response was assessed by investigators every two cycles by computed tomography or magnetic resonance imaging according to Response Evaluation Criteria in Solid Tumors (version 1.1) [23].

Serial blood samples were collected before and after tovorafenib dosing on days 1 and 21 (Q2D dosing) or days 1 and 22 (QW dosing) of cycle 1 for plasma PK analysis. In addition, for patients on Q2D schedules, predose or trough samples were collected on days 9 and 15 (Q2D dosing) or days 8 and 15 (QW dosing) to evaluate time to steady state. A validated liquid chromatography coupled to tandem mass spectroscopy (LC–MS/MS) method was used to quantify plasma concentrations of tovorafenib [24]. The concentrations of tovorafenib were determined using a fully validated bioanalytical method (QPS 96-1116) with a lower limit of quantification at 0.5 ng/mL in plasma. This bioanalytical method used protein precipitation extraction of tovorafenib and its stable labeled internal standard from human plasma with positive ionization mode in mass spectrometry. Plasma concentration–time analysis was performed using noncompartmental analysis. The plasma PK parameters were estimated using a validated version of Phoenix WinNonlin software (Version 6.3 or above, Pharsight Corporation, Raleigh, NC). Terminal half-life was calculated based on the equation: *t*_1/2_ = ln2/*k*_el_ (*k*_el_ = elimination rate constant determined by linear regression analysis of selected time points in the apparent terminal phase of the log plasma concentration versus time curve).

Based on tissue availability, pharmacodynamic assays included assessment of pERK expression levels in paired biopsy samples (baseline and day 21) from patients in the melanoma dose expansion cohorts. The level of staining was assessed both by a pathologist (semi-quantitative measurements according to H-score assessment) and by quantitated image analysis (Aperio, Leica Biosystems Nussloch, Germany).

### Statistical considerations

No formal power calculations were carried out. In the dose escalation phase, enrollment of approximately 54 patients was envisaged, with the actual number dependent on the number of dose escalation steps and the number of patients per cohort. In the dose expansion phase, enrollment of up to 16 patients in each of 8 cohorts was planned, representing an additional 128 patients. Patients were assigned to 1 of the 8 expansion cohorts based on tumor type, mutational status, and/or treatment history (Supplementary Table S1). An interactive voice response system (IVRS) was used to manage patient enrollment, cohort assignment and drug supplies.

The safety population was defined as all patients who received any amount of tovorafenib. The DLT-evaluable population was defined as all patients in the dose escalation phase of the study who either experienced DLT during cycle 1 or who completed at least 75% of the planned doses and had sufficient follow-up data to allow the investigators and sponsor to determine whether DLT had occurred. The response-evaluable population included all patients with measurable disease who received any amount of tovorafenib and who had at least 1 postbaseline response assessment. The PK-evaluable population included all patients who had sufficient dosing data and concentration–time data to permit calculation of PK parameters.

## Results

### Patients and disposition

Between September 13, 2011 and September 5, 2016, 149 patients were enrolled and received at least 1 dose of tovorafenib (safety population). Tovorafenib was administered Q2D to 30 patients in the dose escalation phase and 80 patients in the dose expansion phase (including 20 patients in a PK expansion cohort) and QW to 20 patients in the dose escalation phase and 19 patients in the dose expansion phase. The data cutoff date for the current analysis was April 11, 2017, at which time, a small number of patients remained on treatment. Patient disposition is described in Supplementary Tables S2 and S3. Baseline characteristics of the safety population are summarized in Table 1. The most common primary diagnoses in the dose escalation phase were colon cancer and melanoma. In line with the eligibility requirements, most patients in the dose expansion phase had a primary diagnosis of melanoma. The majority of patients in the dose expansion phase had received 1 or more regimens of prior antineoplastic therapy (68%). Study treatment exposure is summarized in Supplementary Table S4. Patients received a median of 2 cycles of treatment in both the dose escalation and dose expansion phases. The primary reasons for treatment discontinuation were disease progression and adverse events (Supplementary Tables S2 and S3). No patients discontinued treatment because they had a complete response or had completed the maximum number of treatment cycles per protocol.

**Table 1:** Baseline characteristics (safety population)

	Dose escalation phase			Dose expansion phase		
	Q2D	QW	Total	Q2D	QW	Total
	*n* = 30	*n* = 20	*n* = 50	*n* = 80	*n* = 19	*n* = 99
Sex						
Male	14 (47)	9 (45)	23 (46)	43 (54)	10 (53)	53 (54)
Female	16 (53)	11 (55)	27 (54)	37 (46)	9 (47)	46 (46)
Age, years						
Median	65.5	60.5	62.5	65.0	70.0	66.0
Range	37–83	39–74	37–83	31–94	41–83	31–94
Race						
White	26 (87)	18 (90)	44 (88)	78 (98)	19 (100)	97 (98)
Black or African American	3 (10)	2 (10)	5 (10)	0	0	0
Asian	0	0	0	1 (1)	0	1 (1)
Other	1 (3)	0	1 (2)	0	0	0
Not reported	0	0	0	1 (1)	0	1 (1)
Primary diagnosis						
Melanoma	0	4 (20)	4 (8)	62 (78)	19 (100)	81 (82)
Colon	12 (40)	6 (30)	18 (36)	0	0	0
Pancreatic	2 (7)	1 (5)	3 (6)	0	0	0
Thyroid	1 (3)	2 (10)	3 (6)	0	0	0
Other solid tumor	15 (50)	7 (35)	22 (44)	18^a^ (23)	0	18 (18)
Disease stage at study entry						
I	0	1 (5)	1 (2)	0	0	0
II	0	0	0	1 (1)	0	1 (1)
III	0	1 (5)	1 (2)	0	1 (5)	1 (1)
IIIA	0	0	0	1 (1)	0	1 (1)
IIIB	1 (3)	2 (10)	3 (6)	0	2 (11)	2 (2)
IIIC	0	1 (5)	1 (2)	4 (5)	1 (5)	5 (5)
IV	21 (70)	9 (45)	30 (60)	60 (75)	12 (63)	72 (73)
IVA	0	0	0	1 (1)	1 (5)	2 (2)
IVB	1 (3)	2 (10)	3 (6)	2 (3)	0	2 (2)
IVC	1 (3)	1 (5)	2 (4)	5 (6)	2 (11)	7 (7)
NA	6 (20)	3 (15)	9 (18)	6 (8)	0	6 (6)
Prior antineoplastic therapy	NA	19 (95)	19 (38)	52 (65)	15 (79)	67 (68)
Number of prior regimens, *n* (% of treated)						
0	NA	1 (5)	1 (2)	28 (35)	4 (21)	32 (32)
1	NA	2 (11)	2 (11)	15 (29)	3 (20)	18 (27)
2	NA	2 (11)	2 (11)	21 (40)	5 (33)	26 (39)
3	NA	5 (26)	5 (26)	6 (12)	3 (20)	9 (13)
4 or more		10 (53)	10 (53)	10 (19)	4 (27)	14 (21)
Prior radiation therapy	NA	14 (70)	14 (28)	33 (41)	8 (42)	41 (41)
Prior surgery	NA	20 (100)	20 (40)	69 (86)	19 (100)	88 (89)

Data are n (%), unless otherwise stated

DLT dose-limiting toxicity, NA not available, Q2D once every other day, QW once weekly

^a^Other diagnoses in the melanoma Q2D expansion cohorts include choroidal melanoma, ocular melanoma, and uveal melanoma

### Dose escalation and DLTs

In the Q2D dose escalation phase, cohorts of patients received tovorafenib doses of 20 mg, 40 mg, 80 mg, 135 mg, 200 mg and 280 mg Q2D in 22-day cycles, and 200 mg Q2D in 28-day cycles. DLTs occurred in cycle 1 in 2 patients in the 280 mg cohort; 1 patient had grade 3 periorbital edema and 1 had grade 3 maculo-papular rash. The MTD selected for the Q2D expansion cohorts was therefore 200 mg, to be administered over 28-day cycles. In the QW dose escalation phase, cohorts of patients received tovorafenib doses of 400 mg, 600 mg, and 800 mg, in 28-day cycles. DLTs occurred in cycle 1 in 2 patients in the 800 mg cohort; 1 patient had grade 3 hyperbilirubinemia and 1 had grade 3 rash. The MTD selected for the QW expansion cohort was therefore 600 mg to be administered over 28-day cycles.

### Safety and tolerability

The incidence of TEAEs and SAEs is summarized in Supplementary Table S5 and the most common TEAEs are listed in Table 2. Of note, only 1 of 149 treated patients (< 1%; Q2D dose expansion cohort) had squamous cell carcinoma of skin reported as a TEAE. The incidence of drug-related TEAEs according to dosing regimen is summarized in Supplementary Table S6. The two most common in the dose expansion phase were maculo-papular rash in the Q2D cohort (36%) and fatigue (42%) in the QW cohort. In the dose expansion phase, 68% of patients experienced a grade 3 or higher TEAE, including 73% of patients in the Q2D cohorts and 47% in the QW cohort. Grade 3 or higher TEAEs occurring in ≥ 5% of patients are listed in Supplementary Table S7. The two most commonly occurring overall were anemia (14%) and maculo-papular rash (8%).

**Table 2:** Most common treatment-emergent adverse events (safety population)

TEAEs	Dose escalation phase				Dose expansion phase			
	Regardless of attribution			Drug-related^a^	Regardless of attribution			Drug-related^a^
	Q2D, *n* = 30	QW, *n* = 20	Total, *n* = 50	Total, *n* = 50	Q2D, *n* = 80	QW, *n* = 19	Total, *n* = 99	Total, *n* = 99
Any	30 (100)	20 (100)	50 (100)	43 (86)	80 (100)	19 (100)	99 (100)	89 (90)
≥ Grade 3	13 (43)	15 (75)	28 (56)	11 (22)	58 (73)	9 (47)	67 (68)	38 (38)
Preferred term (any grade)								
Fatigue	20 (67)	11 (55)	31 (62)	24 (48)	34 (43)	9 (47)	43 (43)	32 (32)
Anemia	8 (27)	8 (40)	16 (32)	11 (22)	33 (41)	4 (21)	37 (37)	24 (24)
Constipation	9 (30)	4 (20)	13 (26)	5 (10)	30 (38)	4 (21)	34 (34)	15 (15)
Nausea	5 (17)	7 (35)	12 (24)	6 (12)	29 (36)	5 (26)	34 (34)	18 (18)
Rash maculo-papular	11 (37)	2 (10)	13 (26)	13 (26)	30 (38)	3 (16)	33 (33)	32 (32)
Myalgia	6 (20)	5 (25)	11 (22)	10 (20)	20 (25)	3 (16)	23 (23)	17 (17)
Dyspnea	3 (10)	4 (20)	7 (14)	1 (2)	24 (30)	4 (21)	28 (28)	7 (7)
Arthralgia	9 (30)	4 (20)	13 (26)	10 (20)	11 (14)	4 (21)	15 (15)	9 (9)
Vomiting	3 (10)	5 (25)	8 (16)	5 (10)	16 (20)	3 (16)	19 (19)	11 (11)
Edema peripheral	7 (23)	3 (15)	10 (20)	2 (4)	15 (19)	0	15 (15)	4 (4)
Blood creatine phosphokinase increased	0	0	0	0	23 (29)	1 (5)	24 (24)	21 (21)
Pruritus	4 (13)	3 (15)	7 (14)	6 (12)	16 (20)	1 (5)	17 (17)	15 (15)
Decreased appetite	3 (10)	2 (10)	5 (10)	2 (4)	14 (18)	4 (21)	18 (18)	10 (10)
Diarrhea	6 (20)	3 (15)	9 (18)	6 (12)	13 (16)	1 (5)	14 (14)	3 (3)
Headache	3 (10)	6 (30)	9 (18)	4 (8)	11 (14)	2 (11)	13 (13)	5 (5)
Periorbital edema	4 (13)	1 (5)	5 (10)	4 (8)	14 (18)	2 (11)	16 (16)	16 (16)
Pyrexia	2 (7)	2 (10)	4 (8)	0	14 (18)	3 (16)	17 (17)	7 (7)
Dysgeusia	2 (7)	0	2 (4)	2 (4)	16 (20)	1 (5)	17 (17)	13 (13)
Abdominal pain	3 (10)	5 (25)	8 (16)	0	9 (11)	1 (5)	10 (10)	2 (2)
Dermatitis acneiform	4 (13)	0	4 (8)	4 (8)	12 (15)	2 (11)	14 (14)	13 (13)
Pain in extremity	1 (3)	2 (10)	3 (6)	1 (2)	14 (18)	0	14 (14)	3 (3)
Aspartate aminotransferase increased	0	0	0	0	16 (20)	0	16 (16)	10 (10)
Back pain	1 (3)	2 (10)	3 (6)	1 (2)	12 (15)	1 (5)	13 (13)	0
Cough	3 (10)	1 (5)	4 (8)	0	10 (13)	2 (11)	12 (12)	0
Hair color changes	2 (7)	0	2 (4)	1 (2)	13 (16)	1 (5)	14 (14)	12 (12)
Face edema	6 (20)	2 (10)	8 (16)	7 (14)	7 (9)	0	7 (7)	6 (6)

Data are *n* (%). Treatment emergent adverse events shown are those occurring in ≥ 10% of the overall safety population regardless of attribution. The proportion of those events assessed as drug-related is also summarized

CI confidence interval, Q2D once every other day, QW once weekly

^a^Deemed by the site investigator to have had a reasonable possibility of being caused by the study drug

In the Q2D expansion cohorts, drug-related TEAEs of grade 3 or higher occurred in 33 of 80 patients (41%); the most common were maculo-papular rash (9%) and anemia (8%). In the QW expansion cohort, drug-related TEAEs of grade 3 or higher occurred in 4 of 20 patients (20%); the most common was hyperbilirubinemia (10%).

In the dose escalation phase, drug-related treatment-emergent SAEs were reported in 2 of 30 patients (7%) in the Q2D cohort (280 mg dose level; grade 3 anemia in 1 patient, and grade 4 dyspnea and grade 5 respiratory failure in another patient) and 2 of 20 patients (10%) in the QW cohort (800 mg dose level; grade 3 rash, 1 patient, and grade 3 hyperbilirubinemia, 1 patient). In the dose expansion phase, drug-related treatment-emergent SAEs were reported in 12 of 80 patients (15%) in the Q2D cohorts and included acute kidney injury, macular rash, rash maculo-papular (grade 3 events in 2 patients each). In the QW dose expansion cohort, 4 of 19 patients (21%) had drug-related treatment-emergent SAEs, including grade 2 anemia and dyspnea in 1 patient, grade 2 nausea and grade 3 maculo-papular rash in another, and grade 3 erythema multiforme and macular rash in 1 patient each.

In the dose expansion phase, 15 of 80 patients (19%) in the Q2D cohort had TEAEs resulting in permanent discontinuation of tovorafenib. These included maculo-papular rash and sepsis (2 patients [3%] each). In the QW cohort of the dose expansion phase, 4 of 19 patients (21%) had TEAEs resulting in permanent discontinuation, including atrial flutter, dyspnea, erythema multiforme, and fatigue (1 patient each). In the dose expansion phase, 19 of 99 patients (19%) had TEAEs leading to dose reduction including 17 of 80 patients (21%) in the Q2D cohorts and 2 of 19 (11%) in the QW cohort, the most common of which were maculo-papular rash (5 of 99 patients, 5%) and generalized rash (3 patients, 3%).

There were 13 on-study deaths. The fatal SAEs associated with these deaths predominantly related to the underlying disease or complications thereof and are listed in Supplementary Table S8. Only one death, associated with respiratory failure in a patient in the 280 mg Q2D dose escalation cohort, was deemed by the study investigators to be treatment related.

### Response

In the Q2D dose escalation phase, there were no responses in 22 evaluable patients; 5 patients (23%) had a best response of stable disease (Table 3). In the Q2D expansion phase, partial responses were seen in 10 of 68 evaluable patients, representing an objective response rate of 15% (95% CI 7–25; Supplementary Table S9). Responses were seen in 8 of 16 (50%) patients in the *BRAF* mutation-positive, RAF and MEK inhibitor-naïve cohort (cohort 1), 1 of 6 patients (17%) in the *BRAF* mutation-positive RAF and MEK inhibitor-previously treated cohort (cohort 2), and 1 of 14 patients (7%) in the *NRAS* mutation-positive RAF and MEK inhibitor-naïve cohort (cohort 3; Table 3). The overall median duration of response in the 68 evaluable patients was 6.0 months, and median progression-free survival (PFS) was 1.9 months (95% CI 1.8–3.6), with a sustained PFS of 45 months in an individual patient in the *BRAF* mutation-positive, RAF and MEK inhibitor-naïve cohort, who remained in response and on treatment at data cutoff (median PFS in this cohort was 5.7 months; 95% CI 1.9–14.3). One patient with an *NRAS*-mutated melanoma in cohort 7 with demonstrated clinical benefit (42 months with stable disease) continued to receive tovorafenib after the study ended, under a single patient investigational new drug (IND) application. A complete response was reported after 7 months of treatment under this single patient IND, which has been sustained with continued treatment for 8 years [25].

**Table 3:** Response by investigator assessment (response-evaluable population)

	Dose escalation phase		Dose expansion phase: molecularly defined cohorts						
	Q2D *n* = 22	QW *n* = 14	*BRAF*+ Naïve Q2D *n* = 16 Cohort 1	*BRAF*+ Previously treated Q2D *n* = 6 Cohort 2	*NRAS*+ Naïve Q2D *n* = 14 Cohort 3	*NRAS* + Previously treated Q2D *n* = 1 Cohort 4	*BRAF*/*NRAS* WT Naïve Q2D *n* = 6 Cohort 5	*BRAF*/*NRAS* WT Previously Q2D *n* = 9 Cohort 6	*NRAS*+ Naïve QW *n* = 17 Cohort 9
Objective response rate	0	2 (14)	8 (50)	1 (17)	1 (7)	0	0	0	0
95% CI	–	2–43	25–75	< 1–64	< 1–34	–	–	–	–
Best overall response									
Complete response	0	0	0	0	0	0	0	0	0
Partial response	0	2 (14)	8 (50)	1 (17)	1 (7)	0	0	0	0
Stable disease	5 (23)	4 (29)	2 (13)	3 (50)	4 (29)	0	1 (17)	3 (33)	9 (53)
Progressive disease	17 (77)	8 (57)	6 (38)	2 (33)	9 (64)	1 (100)	5 (83)	6 (67)	8 (47)

Data are *n* (%), unless otherwise stated

*BRAF*+ *BRAF* mutation-positive, DLT dose-limiting toxicity, *NRAS*+  *NRAS* mutation-positive, Q2D once every other day, QW once weekly, WT wild-type

In the QW dose escalation phase, there were 2 partial responses in 14 evaluable patients (14%); 1 patient with endometrial cancer at the 600 mg dose level and 1 patient with thyroid cancer at the 800 mg dose level. The *KRAS*, *BRAF*, and *NRAS* mutation status of the tumors in these 2 patients was unknown or not reported. In the QW dose expansion phase, there were no responses in 17 evaluable patients; 9 patients (53%) had a best response of stable disease.

**Fig. 1:**
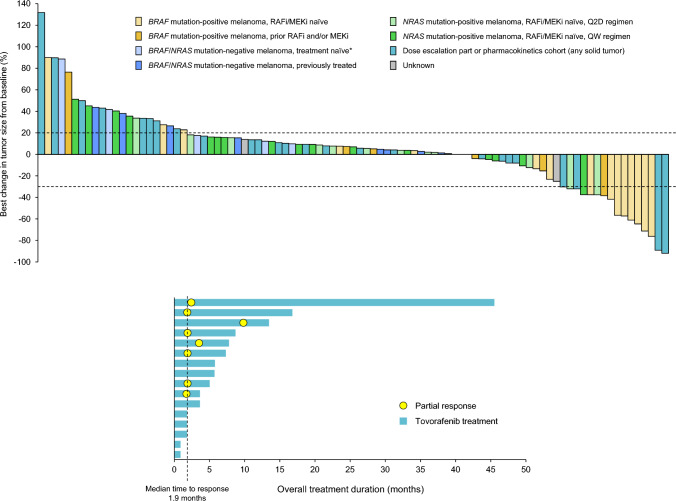
**A** Best tumor response in 93 evaluable patients in the dose escalation and expansion phases. In the dose expansion cohorts, tumor size data were missing for 4 response-evaluable patients. *Except for prior ipilimumab, PD-1, or PD-L1 monoclonal antibody therapy. **B** Time on treatment and timing of response for patients in cohort 1 with *BRAF* mutation-positive melanoma naïve to RAF and MEK inhibitors

Best tumor response from baseline in 93 evaluable study patients is shown in Fig. 1A and time on treatment and timing of response for the 16 patients in cohort 1 is summarized in Fig. 1B.

### Pharmacokinetics

**Fig. 2:**
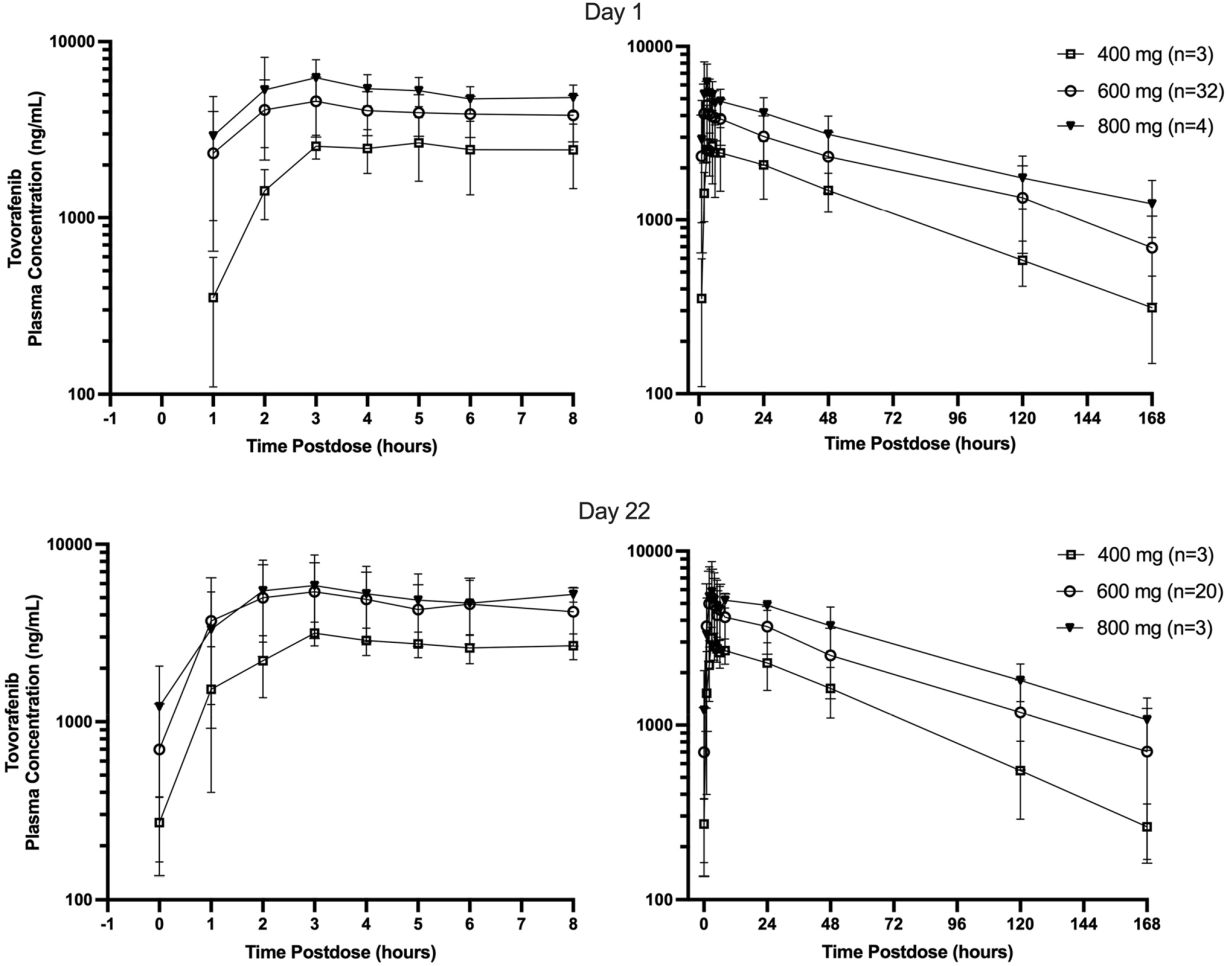
Mean (± standard deviation) plasma concentration–time profiles of tovorafenib on days 1 and 22 following once weekly oral administration

Mean (± standard deviation) plasma concentration–time profiles of tovorafenib by QW dose group on days 1 and 22 of cycle 1 are shown in Fig. 2; cycle 1 day 22 plasma PK parameters are summarized by dose group in Table 4. Following multiple oral dosing of 600 mg QW, peak concentrations of tovorafenib were achieved at a median *T*_max_ of 3 h post-dose (range 1–24 h) on cycle 1 day 22. Minimal to no apparent accumulation in terms of day 22 AUC_168_ over day 1 AUC_168_ was observed following repeated QW dosing. The mean plasma terminal half-life (*t*_1/2_) of tovorafenib was approximately 70 h (range 31–119 h) as defined in 20 evaluable patients receiving 600 mg QW. The relationship between dose and cycle 1 day 22 tovorafenib exposures (AUC_168_) is shown in Supplementary Figure S1. Steady-state exposures increased in an approximately dose-proportional manner over the 400 mg to 800 mg QW dose range with the 95% CI of the power model containing 1 (95% CI 0.55–2.04), with the coefficient of 1.30. For QW dosing regimens, minimum drug accumulation was observed and the geometric mean *R*_auc_ (accumulation ratio based on AUC_0-last_) was in the range of 1.03–1.09. With the Q2D dosing regimen at 200 mg, the geometric mean value of *R*_auc_ was ~ 2.55.

**Table 4:** Plasma pharmacokinetic parameters of tovorafenib on cycle 1 day 22 following weekly oral administration

	*n*	*C*_max_ (ng/mL) Geometric mean (%CV^b^)	*T*_max_^c^ (h) Median (min, max)	AUC_168_ (ng*h/mL) Geometric mean (%CV)	*t*_1/2z_ (h) Mean (range)	*R*_auc_^d^ Mean (SD)
400 mg (*n* = 3)	3	3140 (15)	3 (3, 3)	193,000 (29)	52.0 (46.4–59.3)	1.08 (0.18)
600 mg (*n* = 32)^a^	20	5650 (36)	3 (1, 24)	330,000 (33)	70.5 (31.1–119)	1.09^e^ (0.24)
800 mg (*n* = 3)	3	6460 (25)	4 (3, 7)	473,000 (18)	68.1 (61.6–73.0)	1.03 (0.19)

%CV percentage coefficient of variation, AUC_168_ area under the plasma concentration versus time curve from 0 to 168 h postdose, *C*_max_ maximum observed plasma concentration, max maximum, min minimum, *R*_auc_ accumulation ratio based on AUC_168,_ Q2D once every other day, QW once weekly, SD standard deviation, *t*_1/2z_ terminal disposition phase half-life, *T*_max_ time to reach peak plasma concentration

^a^Comprising 600 mg QW 28 days dose escalation cohort (*n* = 13) and *NRAS* mutation-positive, naïve to prior therapy with RAF and MEK inhibitors QW expansion cohort (*n* = 19)

^b^%CV was calculated (100*mean/SD)

^c^*T*_max_ = time to reach observed peak blood/plasma concentration

^d^Accumulation ratio was calculated based on AUC values

^e^*n* = 19

Note: the data in this table have been rounded for clarity

Similar PK analyses were carried out by Q2D dose group (Supplementary Figs. S1 and S2, and Supplementary Table S10). Steady-state tovorafenib AUC_48_ increased in an approximately dose-proportional manner over the dose ranges of 20 mg to 280 mg Q2D. While no apparent accumulation was observed with the QW dose regimens, Q2D administration resulted in approximately 2.5-fold accumulation in AUC_48_ at steady state.

### Pharmacodynamic assessments

In general, the median level of pERK staining in evaluable sample pairs from each of 5 melanoma Q2D expansion cohorts (Supplementary Table S3), was lower at day 21 than baseline as assessed by H-score by a pathologist (median percentage decrease ≥ 70% in the *BRAF* mutation-positive treatment-naïve cohort, *BRAF* mutation-positive previously treated cohort and *NRAS* mutation-positive treatment-naïve cohort) and by quantitated image analysis (median percentage decrease ≥ 70% in the *BRAF* mutation-positive previously treated cohort and *NRAS* mutation-positive treatment-naïve cohort), indicative of inhibition of RAF signaling. In the QW melanoma expansion cohort, the median level of pERK expression as assessed by both methods had decreased slightly by day 21 (median percentage decreases 12% and 8%, respectively).

### Recommended phase 2 dose

Based on all available data including safety, efficacy and PK, the RP2Ds of tovorafenib in patients with relapsed or refractory solid tumors were deemed to be 200 mg Q2D and 600 mg QW.

## Discussion

This first-in-human dose escalation study allowed the determination of the MTD of tovorafenib administered either Q2D or QW and has shown that the overall safety profiles on both schedules are acceptable. The most common TEAEs leading to dose reduction were skin and subcutaneous tissue disorders, which have previously been noted as common side effects of first-generation BRAF inhibitors [26]. In line with preclinical data suggesting that tovorafenib does not trigger paradoxical activation of ERK signaling, squamous cell carcinoma of the skin was reported as a TEAE in only 1 (< 1%) of 149 treated patients. By contrast, such lesions have been reported to occur relatively frequently in patients treated with first-generation BRAF inhibitors [26]. Overall, there were 13 on-study deaths (12 fatal SAEs, and 1 patient who died of gastric cancer not reported as an SAE) with only one, respiratory failure in a patient in the 280 mg Q2D dose escalation cohort, deemed by the site investigator to be related to the study drug.

The dose expansion phase provided a preliminary indication of tovorafenib efficacy. Partial responses were seen in 8 (50%) of 16 patients in the *BRAF* mutation-positive, RAF and MEK inhibitor-naïve cohort who received the Q2D RP2D. This level of monotherapy activity is in line with that seen in phase 1 studies of first-generation agents in a similar setting [27, 28].

The PK analyses showed that tovorafenib has a moderately fast absorption rate, with an overall median *T*_max_ of 2–4 h post-dose. Overall mean accumulation following 21 days of Q2D dosing was 2.5-fold. By contrast, QW dose administration was associated with minimal to no apparent accumulation of tovorafenib in systemic circulation in the dose range of 400 mg to 800 mg. Steady-state AUC increased in an approximately dose-proportional manner for both Q2D and QW dose ranges tested. The plasma terminal half-life (*t*_1/2_) of tovorafenib was approximately 70 h.

The QW dose escalation and expansion cohorts were introduced by protocol amendment as it was anticipated that higher unit doses would be possible on such a schedule, which would lead to higher tovorafenib concentrations for part of the treatment period. This proved to be the case, with a higher *C*_max_ value reached for the QW MTD compared with the Q2D MTD. Preliminary exposure–response analysis using data from both dosing regimens supported the selection of QW dosing for future clinical development as modeling and simulation results indicated that the marginal increase in efficacy associated with more frequent dosing (e.g., Q2D) was outweighed by an increase in the incidence of grade 3 rash along with other findings from exposure-adverse event and exposure-safety biomarker analyses [29, 30].

Weekly administration of tovorafenib as monotherapy has been further explored in a pediatric phase 1 study in patients with radiographically recurrent/progressive low-grade gliomas (LGGs) harboring MAPK pathway alterations [31]. In the initial dose-escalation part of this study, tovorafenib was well tolerated and of 8 patients with tumor *RAF* gene fusions, 2 had complete responses, 3 had partial responses and two achieved prolonged stable disease (NCT03429803). In the phase 1b part of this study, tovorafenib demonstrated clinically meaningful activity in 24 (69%) of 35 patients with MAPK pathway-altered cancers (2 complete responses, 7 partial responses and 15 stable diseases) [32]. Tovorafenib QW monotherapy is also being investigated in the pivotal phase 2 FIREFLY-1 study in patients aged 6 months–25 years with relapsed or progressive LGGs harboring *BRAF* alterations, including *BRAF* fusions and *BRAF* mutations (NCT04775485). An interim analysis of the first 25 enrolled patients with ≥ 6 months of follow-up showed encouraging antitumor activity with an overall response rate of 64% and a clinical benefit rate of 91%. Tovorafenib was generally well tolerated, with most adverse events being grade 1 or 2 [33].

Tovorafenib on a QW schedule is also currently being evaluated as monotherapy and in combination with other therapies in the phase 1b/2 FIRELIGHT-1 umbrella study in patients ≥ 12 years of age with recurrent, progressive, or refractory solid tumors harboring MAPK pathway aberrations (NCT04985604). In particular, given non-overlapping toxicity profiles, this study will explore combining tovorafenib with a MEK inhibitor, which outside the specific setting of tumors with *RAF* fusions, may be a more effective treatment approach than tovorafenib monotherapy in patients with tumors harboring other MAPK pathway alterations. Further, the randomized phase 3 LOGGIC/FIREFLY-2 study will evaluate the efficacy, safety, and tolerability of tovorafenib QW monotherapy versus standard of care chemotherapy in children and young adults with LGGs harboring an activating *RAF* alteration and requiring front-line systemic therapy (NCT05566795).

In conclusion, we have defined the MTD of tovorafenib for adults on Q2D and QW schedules. The dose expansion phase of our phase 1 study shows that the safety profile of tovorafenib is acceptable in both cases, and in line with other BRAF-targeted agents. Of note, tovorafenib appears to have antitumor activity in the setting of BRAF alterations without the clinical manifestations of paradoxical activation seen with type I BRAF inhibitors, such as the development of cutaneous squamous cell carcinoma or keratoacanthoma. In addition, there is evidence of MAPK pathway inhibition without the class effects seen with MEK inhibitors (e.g., decreased left ventricular ejection fraction, retinal vein obstruction/central serous retinopathy, acneiform rash, paronychia).The long plasma half-life of tovorafenib affords use with a QW dosing schedule, while still maintaining a steady state trough plasma concentration above the protein binding adjusted pERK EC_50_ inhibition level. The preliminary indication of antitumor activity in *BRAF*-mutated melanoma is promising although further clinical development of single agent use in this setting in tumors that do not harbor *RAF* fusions (e.g., those with *KRAS* or *NRAS* mutations) is likely to be limited. However, tovorafenib in combination other MAPK pathway and non-MAPK pathway targeted agents should be further explored, with emerging data justifying continued clinical development across multiple settings.

### Supplementary Information

Below is the link to the electronic supplementary material.

Supplementary file1 (DOCX 320 KB)

## Data Availability

Study data are available within the ClinicalTrials.gov NCT01425008 record: (https://clinicaltrials.gov/ct2/show/results/NCT01425008).
