# Supplementary material for: Phase 1 study of the pan-RAF inhibitor tovorafenib in patients with advanced solid tumors followed by dose expansion in patients with metastatic melanoma
Source: Cancer Chemother Pharmacol. 2023 May 23;92(1):15–28. doi: 10.1007/s00280-023-04544-5 (PMC10261210; doi:10.1007/s00280-023-04544-5)
Supplement: Supplementary file 1 — Supplementary file1 (DOCX 320 KB) [file 280_2023_4544_MOESM1_ESM.docx]

# **SUPPLEMENTARY APPENDIX**

Supplement to: Phase 1 study of the pan-RAF inhibitor tovorafenib in patients with advanced solid tumors followed by dose expansion in patients with metastatic melanoma

*Cancer Chemotherapy and Pharmacology*

Drew W. Rasco, Theresa Medina, Pippa Corrie, Anna C. Pavlick, Mark R. Middleton, Paul Lorigan, Chris Hebert, Ruth Plummer, James Larkin, Sanjiv S. Agarwala, Adil I. Daud, Jiaheng Qiu, Viviana Bozon, Michelle Kneissl, Elly Barry,* Anthony J. Olszanski

*Correspondence: Elly Barry MD MMSc

Day One Biopharmaceuticals

2000 Sierra Point Parkway, Suite 501

Brisbane

CA 94005

USA

email: elly.barry@dayonebio.com

**Table S1.** Eligibility criteria for dose expansion cohorts

| **Cohort** | **Eligibility criteria** |
| --- | --- |
| Q2D |  |
| 1 | *BRAF* mutation-positive cutaneous melanoma, naïve to prior therapy with  RAF and MEK inhibitors |
| 2 | *BRAF* mutation-positive cutaneous melanoma, which in response to previous  treatment with RAF inhibitors and/or MEK inhibitors, had:   - relapsed following an objective response - failed to demonstrate an objective response, and/or - the patient could not tolerate such a regimen due to unacceptable toxicity |
| 3 | *NRAS* mutation-positive cutaneous melanoma, naïve to prior therapy with  RAF and MEK inhibitors |
| 4 | *NRAS* mutation-positive cutaneous melanoma, which in response to previous  treatment with MEK inhibitors, had:   - relapsed following an objective response - failed to demonstrate an objective response, and/or - the patient could not tolerate such a regimen due to unacceptable toxicity |
| 5 | *BRAF*/*NRAS* mutation-negative cutaneous melanoma (wild-type), naïve to  any prior anticancer therapy except ipilimumab, PD-1, or PD-L1 monoclonal antibodies |
| 6 | *BRAF*/*NRAS* mutation-negative melanoma (wild-type), received at least 1 line  of prior anticancer therapy. Cutaneous, uveal, or mucosal melanoma permitted in this cohort |
| 7 | PK cohort: Patients with any advanced solid tumor (excluding lymphoma, but including melanoma) who had failed or were not candidates for standard therapies or for whom no approved therapy was available |
| QW |  |
| 8 | *BRAF* mutation-positive cutaneous melanoma (note: enrollment in this cohort was not initiated) |
| 9 | *NRAS* mutation-positive cutaneous melanoma, naïve to prior therapy with  RAF and MEK inhibitors |

Abbreviations: PK, pharmacokinetics; Q2D, once every other day; QW, once weekly.

**Table S2.** Patient disposition: dose escalation phase

| **Dose level**  **Schedule**  **Cycle length**  **n** | **20 mg**  **Q2D**  **22 days**  **(n=4)** | **40 mg**  **Q2D**  **22 days**  **(n=3)** | **80 mg**  **Q2D**  **22 days**  **(n=3)** | **135 mg**  **Q2D**  **22 days**  **(n=3)** | **200 mg**  **Q2D**  **22 days**  **(n=7)** | **280 mg**  **Q2D**  **22 days**  **(n=7)** | **200 mg**  **Q2D**  **28 days**  **(n=3)** | **Q2D**  **Total**  **(n=30)** | **400 mg**  **QW**  **28 days**  **(n=3)** | **600 mg**  **QW**  **28 days**  **(n=13)** | **800 mg**  **QW**  **28 days**  **(N=4)** | **QW**  **Total**  **(n=20)** | **Total**  **(n=50)** |
| --- | --- | --- | --- | --- | --- | --- | --- | --- | --- | --- | --- | --- | --- |
| Study populations |  |  |  |  |  |  |  |  |  |  |  |  |  |
| Safety | 4 | 3 | 3 | 3 | 7 | 7 | 3 | 30 | 3 | 13 | 4 | 20 | 50 |
| DLT-evaluable | 4 (100) | 3 (100) | 3 (100) | 3 (100) | 6 (86) | 6 (86) | 3 (100) | 28 (93) | 3 (100) | 10 (77) | 4 (100) | 17 (85) | 45 (90) |
| Response evaluable | 3 (75) | 3 (100) | 2 (67) | 3 (100) | 6 (86) | 4 (57) | 1 (33) | 22 (73) | 3 (100) | 8 (62) | 3 (75) | 14 (70) | 36 (72) |
| PK evaluable | 4 (100) | 3 (100) | 3 (100) | 3 (100) | 7 (100) | 7 (100) | 3 (100) | 30 (100) | 3 (100) | 13 (100) | 4 (100) | 20 (100) | 50 (100) |
| Primary reason for discontinuation of study treatment |  |  |  |  |  |  |  |  |  |  |  |  |  |
| Progressive disease | 2 (50) | 3 (100) | 2 (67) | 3 (100) | 5 (71) | 4 (57) | 0 | 19 (63) | 2 (67) | 6 (46) | 2 (50) | 10 (50) | 29 (58) |
| Adverse event | 2 (50) | 0 | 0 | 0 | 0 | 2 (29) | 0 | 4 (13) | 0 | 2 (15) | 0 | 2 (10) | 6 (12) |
| Symptomatic deterioration | 0 | 0 | 1 (33) | 0 | 0 | 0 | 0 | 1 (3) | 0 | 2 (15) | 2 (50) | 4 (20) | 5 (10) |
| Other | 0 | 0 | 0 | 0 | 0 | 0 | 0 | 0 | 0 | 1 (8) | 0 | 1 (5) | 1 (2) |
| Withdrawal by patient | 0 | 0 | 0 | 0 | 1 (14) | 1 (14) | 1 (33) | 3 (10) | 0 | 1 (8) | 0 | 1 (5) | 4 (8) |
| Unsatisfactory therapeutic response | 0 | 0 | 0 | 0 | 1 (14) | 0 | 2 (67) | 3 (10) | 1 (33) | 1 (8) | 0 | 2 (10) | 5 (10) |

Data are n (%).

Abbreviations: DLT, dose-limiting toxicity; PK, pharmacokinetics; Q2D, once every other day; QW, once weekly.

**Table S3.** Patient disposition: dose expansion phase

|  | ***BRAF*+**  **Naïve**  **(n=16)**  Cohort 1 | ***BRAF*+**  **Previously**  **treated**  **(n=8)**  Cohort 2 | ***NRAS*+**  **Naïve**  **Q2D**  **(n=16)**  Cohort 3 | ***NRAS*+**  **Previously**  **treated**  **(n=1)**  Cohort 4 | ***BRAF*/*NRAS***  **WT**  **Naïve**  **(n=6)**  Cohort 5 | ***BRAF*/*NRAS***  **WT**  **Previously**  **treated**  **(n=11)**  Cohort 6 | **PK**  **cohort**  **(n=20)**  Cohort 7 | **Unknown**  **(n=2)** | **Total**  **Q2D**  **(n=80)** | ***NRAS*+**  **Naïve**  **QW**  **(n=19)**  Cohort 9 | **Total**  **(n=99)** |
| --- | --- | --- | --- | --- | --- | --- | --- | --- | --- | --- | --- |
| Study populations |  |  |  |  |  |  |  |  |  |  |  |
| Safety | 16 | 8 | 16 | 1 | 6 | 11 | 20 | 2 | 80 | 19 | 99 |
| Response evaluable | 16 (100) | 6 (75) | 14 (88) | 1(100) | 6 (100) | 9 (82) | 14 (70) | 2 (100) | 68 (85) | 17 (89) | 85 (86) |
| PK evaluable | 12 (75) | 3 (38) | 15 (94) | 0 | 1 (17) | 5 (45) | 20 (100) | 0 | 56 (70) | 19 (100) | 75 (76) |
| Pharmacodynamics evaluable | 3 (19) | 4 (50) | 6 (38) | 0 | 2 (33) | 5 (45) | 0 | 1 (50) | 21 (26) | 3 (16) | 24 (24) |
| Primary reason off study treatment |  |  |  |  |  |  |  |  |  |  |  |
| Progressive disease | 10 (63) | 4 (50) | 12 (75) | 1 (100) | 5 (83) | 8 (73) | 9 (45) | 2 (100) | 51 (64) | 10 (53) | 61 (62) |
| Adverse event | 2 (13) | 3 (38) | 3 (19) | 0 | 1 (17) | 1 (9) | 5 (25) | 0 | 15 (19) | 3 (16) | 18 (18) |
| Symptomatic deterioration | 2 (13) | 1 (13) | 0 | 0 | 0 | 1 (9) | 2 (10) | 0 | 6 (8) | 1 (5) | 7 (7) |
| Other | 0 | 0 | 1 (6) | 0 | 0 | 0 | 2 (10) | 0 | 3 (4) | 4 (21) | 7 (7) |
| Withdrawal by patient | 1 (6) | 0 | 0 | 0 | 0 | 1 (9) | 1 (5) | 0 | 3 (4) | 0 | 3 (3) |
| Unsatisfactory therapeutic response | 0 | 0 | 0 | 0 | 0 | 0 | 0 | 0 | 0 | 1 (5) | 1 (1) |
| Lost to follow-up | 0 | 0 | 0 | 0 | 0 | 0 | 1 (5) | 0 | 1 (1) | 0 | 1 (1) |

Data are n (%).
Enrollment into Cohort 8 was not initiated and was deleted from the protocol in a subsequent amendment.

Abbreviations: *BRAF*+, *BRAF* mutation-positive; DLT, dose-limiting toxicity; *NRAS*+, *NRAS* mutation-positive; PK, pharmacokinetics; Q2D, once every other day; QW, once weekly; WT, wild-type.

**Table S4.** Tovorafenib exposure (safety population)

|  | **Dose escalation phase** | | | **Dose expansion phase** | | |
| --- | --- | --- | --- | --- | --- | --- |
|  | **Q2D**  **n=30** | **QW**  **n=20** | **Total**  **n=50** | **Q2D**  **n=80^a^** | **QW**  **n=19** | **Total**  **n=99** |
| Number of cycles, n^b^  Median  Range | 2.0  1–38 | 1.0  1–10 | 2.0  1–38 | 2.0  1–49 | 2.0  1–8 | 2.0  1–49 |
| Total amount of dose received, mg  Median  Range | 2800.0  180–94640 | 2400.0  600–15600 | 2600.0  180–94640 | 5600.0  400–95340 | 4800.0  1200–17400 | 5400.0  400–95340 |
| Total number of doses received, n  Median  Range | 20.0  2–471 | 4.0  1–39 | 11.0  1–471 | 28.0  2–675 | 8.0  2–29 | 25.0  2–675 |
| Relative dose intensity, %  Median  Range | 95.5  18–100 | 75.0  25–100 | 90.9  18–100 | 88.6  18–100 | 87.5  50–100 | 88.0  18–100 |

^a^Two patients in the Q2D total group of the dose expansion phase switched to weekly dosing starting with cycles 4 and 5, respectively.

^b^A cycle was defined as those in which the patient received any amount of study drug.

Abbreviations: Q2D, once every other day; QW, once weekly.

**Table S5.** Overview of TEAE and SAE incidence (safety population)

|  | **Dose escalation phase** | | | **Dose expansion phase** | | |
| --- | --- | --- | --- | --- | --- | --- |
|  | **Q2D**  **n=30** | **QW**  **n=20** | **Total**  **n=50** | **Q2D**  **n=80** | **QW**  **n=19** | **Total**  **n=99** |
| TEAEs | 30 (100) | 20 (100) | 50 (100) | 80 (100) | 19 (100) | 99 (100) |
| ≥Grade 3 | 13 (43) | 15 (75) | 28 (56) | 58 (73) | 9 (47) | 67 (68) |
| Related to study drug^a^ | 26 (87) | 17 (85) | 43 (86) | 72 (90) | 17 (89) | 89 (90) |
| Related ≥grade 3 | 7 (23) | 4 (20) | 11 (22) | 33 (41) | 5 (26) | 38 (38) |
| Leading to discontinuation | 4 (13) | 2 (10) | 6 (12) | 15 (19) | 4 (21) | 19 (19) |
| Leading to drug reduction | 1 (3) | 1 (5) | 2 (4) | 17 (21) | 2 (11) | 19 (19) |
| Leading to dose modification (delay, reduction, discontinuation) | 6 (20) | 4 (20) | 10 (20) | 30 (38) | 7 (37) | 37 (37) |
| SAEs | 10 (33) | 10 (50) | 20 (40) | 40 (50) | 8 (42) | 48 (48) |
| Related to study drug^a^ | 2 (7) | 2 (10) | 4 (8) | 12 (15) | 4 (21) | 16 (16) |

Data are n (%).

^a^Deemed by the site investigator to have had a reasonable possibility of being caused by the study drug.

Abbreviations: Q2D, once every other day; QW, once weekly; TEAE, treatment-emergent adverse events; SAEs, serious adverse events.

**Table S6.** Most common drug-related^a^ treatment-emergent adverse events (safety population)

| Preferred term | **Dose escalation phase** | | | **Dose expansion phase** | | |
| --- | --- | --- | --- | --- | --- | --- |
|  | **Q2D**  **n=30** | **QW**  **n=20** | **Total**  **n=50** | **Q2D**  **n=80** | **QW**  **n=19** | **Total**  **n=99** |
| Fatigue | 15 (50) | 9 (45) | 24 (48) | 24 (30) | 8 (42) | 32 (32) |
| Rash maculo-papular | 11 (37) | 2 (10) | 13 (26) | 29 (36) | 3 (16) | 32 (32) |
| Anemia | 5 (17) | 6 (30) | 11 (22) | 21 (26) | 3 (16) | 24 (24) |
| Myalgia | 6 (20) | 4 (20) | 10 (20) | 14 (18) | 3 (16) | 17 (17) |
| Nausea | 3 (10) | 3 (15) | 6 (12) | 16 (20) | 2 (11) | 18 (18) |
| Blood creatine phosphokinase increased | 0 | 0 | 0 | 20 (25) | 1 (5) | 21 (21) |
| Pruritus | 4 (13) | 2 (10) | 6 (12) | 14 (18) | 1 (5) | 15 (15) |
| Constipation | 3 (10) | 2 (10) | 5 (10) | 13 (16) | 2 (11) | 15 (15) |
| Periorbital edema | 3 (10) | 1 (5) | 4 (8) | 14 (18) | 2 (11) | 16 (16) |
| Arthralgia | 7 (23) | 3 (15) | 10 (20) | 6 (8) | 3 (16) | 9 (9) |
| Dermatitis acneiform | 4 (13) | 0 | 4 (8) | 11 (14) | 2 (11) | 13 (13) |
| Vomiting | 1 (3) | 4 (20) | 5 (10) | 8 (10) | 3 (16) | 11 (11) |
| Dysgeusia | 2 (7) | 0 | 2 (4) | 12 (15) | 1 (5) | 13 (13) |
| Face edema | 6 (20) | 1 (5) | 7 (14) | 6 (8) | 0 | 6 (6) |
| Hair color changes | 1 (3) | 0 | 1 (2) | 11 (14) | 1 (5) | 12 (12) |
| Decreased appetite | 1 (3) | 1 (5) | 2 (4) | 7 (9) | 3 (16) | 10 (10) |
| Aspartate aminotransferase increased | 0 | 0 | 0 | 10 (13) | 0 | 10 (10) |
| Diarrhea | 4 (13) | 2 (10) | 6 (12) | 2 (3) | 1 (5) | 3 (3) |
| Headache | 2 (7) | 2 (10) | 4 (8) | 3 (4) | 2 (11) | 5 (5) |
| Photosensitivity reaction | 0 | 0 | 0 | 7 (9) | 2 (11) | 9 (9) |
| Blood bilirubin increased | 0 | 0 | 0 | 7 (9) | 1 (5) | 8 (8) |
| Dry mouth | 0 | 1 (5) | 1 (2) | 7 (9) | 0 | 7 (7) |
| Dry skin | 1 (3) | 0 | 1 (2) | 5 (6) | 2 (11) | 7 (7) |
| Dyspnea | 1 (3) | 0 | 1 (2) | 5 (6) | 2 (11) | 7 (7) |
| Flushing | 3 (10) | 0 | 3 (6) | 3 (4) | 2 (11) | 5 (5) |
| Rash macular | 0 | 1 (5) | 1 (2) | 6 (8) | 1 (5) | 7 (7) |

Data are n (%). Drug-related treatment emergent adverse events shown are those occurring in ≥5% of the overall population.

^a^Deemed by the site investigator to have had a reasonable possibility of being caused by the study drug.

Abbreviations: Q2D, once every other day; QW, once weekly.

**Table S7.** Grade 3 or higher treatment-emergent adverse events occurring in ≥5% of patients

|  | **Dose escalation phase** | | | **Dose expansion phase** | | |
| --- | --- | --- | --- | --- | --- | --- |
|  | **Q2D**  **n=30** | **QW**  **n=20** | **Total**  **n=50** | **Q2D**  **n=80** | **QW**  **n=19** | **Total**  **n=99** |
| Anemia | 4 (13) | 2 (10) | 6 (12) | 12 (15) | 2 (11) | 14 (14) |
| Rash maculo-papular | 1 (3) | 0 | 1 (2) | 7 (9) | 1 (5) | 8 (8) |
| Dyspnea | 1 (3) | 3 (15) | 4 (8) | 3 (4) | 1 (5) | 4 (4) |
| Fatigue | 3 (10) | 1 (5) | 4 (8) | 3 (4) | 1 (5) | 4 (4) |

Data are n (%).

Abbreviations: Q2D, once every other day; QW, once weekly.

**Table S8.** Fatal serious adverse events (safety population)

| **Patient age, years** | **Dose level/schedule** | **MedDRA preferred term** | **Study day of death** | **Investigator-assessed relationship to study drug** | **Comments** |
| --- | --- | --- | --- | --- | --- |
| Dose escalation phase |  |  |  |  |  |
| 83 | 20 mg Q2D | Respiratory failure | 23 | Not related | Related to disease under study^a^ |
| 68 | 80 mg Q2D | Colon cancer | 44 | Not related | Related to disease under study^a^ |
| 65 | 280 mg Q2D | Respiratory failure | 28 | Related | Respiratory failure occurred in the setting of  both progressive pulmonary metastases and interstitial changes on chest X-ray |
| 71 | 600 mg QW | Pancreatic carcinoma | 28 | Not related | Related to disease under study^a^ |
| 50 | 600 mg QW | Colon cancer metastatic | 9 | Not related | Related to disease under study^a^ |
| 65 | 800 mg QW | Cardio-respiratory arrest | 31 | Not related | Related to disease under study^a^ |
| Dose expansion phase^b^ |  |  |  |  |  |
| 68 | Q2D | Metastatic malignant melanoma | 61 | Not related | Related to disease under study^a^ |
| 37 | Q2D | Sepsis | 26 | Not related | Related to disease under study^a^ |
| 80 | Q2D | Pneumonia | 43 | Not related | Related to disease under study^a^ |
| 57 | Q2D | Malignant melanoma | 67 | Not related | Related to disease under study^a^ |
| 65 | Q2D | Small intestinal obstruction | 16 | Not related | Related to disease under study^a^ |
| 50 | Q2D | Metastatic malignant melanoma | 81 | Not related | Related to disease under study^a^ |

On-study death was defined as a death that occurred between the first dose of study drug and 30 days of the last dose of study drug.

^a^Or complications thereof.

^b^There was 1 additional on-study death in a patient from the Q2D PK expansion cohort, which was reported in the safety database, but was not captured in the clinical database. Therefore, on-study deaths were reported in a total of 7 patients (7%) from the dose expansion phase.

Abbreviations: MedDRA, Medical Dictionary for Regulatory Activities; Q2D, once every other day; QW, once weekly.

**Table S9.** Response by investigator assessment (response-evaluable population)

|  | **Dose escalation phase** | | | **Dose expansion phase** | | |
| --- | --- | --- | --- | --- | --- | --- |
|  | **Q2D**  **n=22** | **QW**  **n=14** | **Total**  **n=36** | **Q2D***  **n=68** | **QW**  **n=17** | **Total**  **n=85** |
| Objective response rate | 0 | 2 (14) | 2 (6) | 10 (15) | 0 | 10 (12) |
| 95% CI | - | 2–43 | <1–19 | 7–25 | - | 6–21 |
| Best overall response |  |  |  |  |  |  |
| Complete response | 0 | 0 | 0 | 0 | 0 | 0 |
| Partial response | 0 | 2 (14) | 2 (6) | 10 (15) | 0 | 10 (12) |
| Stable disease | 5 (23) | 4 (29) | 9 (25) | 21 (31) | 9 (53) | 30 (35) |
| Progressive disease | 17 (77) | 8 (57) | 25 (69) | 37 (54) | 8 (47) | 45 (53) |

Data are n (%).

*Includes response-evaluable patients in molecularly defined melanoma cohorts (n=52), PK cohort (n=14) and unknown (n=2).

Abbreviations: PK, pharmacokinetics; Q2D, once every other day; QW, once weekly.

**Table S10.** Plasma pharmacokinetic parameters of tovorafenib on cycle 1 day 21 following every other day oral administration

|  | **n** | **C_max_**  **(ng/mL)**  **Geometric mean**  **(%CV)** | **t_max_**  **(h)**  **Median**  **(min, max)** | **AUC_48_**  **(ng*h/mL)**  **Geometric mean**  **(%CV)** | **Accumulation ratio^b^**  **Mean**  **(SD)** |
| --- | --- | --- | --- | --- | --- |
| 20 mg (n=4) | 3 | 302  (20) | 2  (2, 4) | 10400  (24) | 3.06  (0.52) |
| 40 mg (n=3) | 2 | 760  (18) | 3  (2, 4) | 20900  (17) | 1.96  (0.47) |
| 80 mg (n=3) | 3 | 2040  (49) | 4  (2, 6) | 42700^b^  (24) | 2.09^c^  (0.53) |
| 135 mg (n=3) | 3 | 3320  (36) | 2  (1, 2) | 96800  (29) | 2.75  (0.20) |
| 200 mg (n=7) | 6 | 3810  (12) | 3  (1, 8) | 118000^c^  (37) | 2.75^d^  (1.09) |
| 200 mg (n=23)^a^ | 14 | 3680  (33) | 2  (2, 23) | 127000  (29) | 2.5  (0.62) |
| 280 mg (n=7) | 4 | 4060  (19) | 3  (2, 4) | 155000^d^  (17) | 2.50^e^  (0.39) |

^a^200 mg pharmacokinetics expansion cohort (n=20) and 200 mg cohort with a 28-day cycle (n=3).

^b^The accumulation ratio was calculated based on AUC values.

^c^n=2.

^d^n=5.

^e^n=3.

Note: the data in this table have been rounded for clarity.

Abbreviations: %CV, percentage coefficient of variation; AUC_48_, area under the plasma concentration versus time curve from 0 to 48 hours postdose; C_max_, maximum observed plasma concentration; max, maximum; SD, standard deviation; t_max_, first time to C_max_.

**A**

**B**

**Figure S1.**

Analysis of the relationship between tovorafenib (TAK-580) Q2D dose and steady-state exposures (AUC_48_; n=32, **A**) and QW dose and steady-state exposures (AUC_168_; n=26, **B**).

**Figure S2.**

Mean (±SD plasma concentration-time profiles of tovorafenib on days 1 and 21 following every other day administration. Data from the 200 mg PK expansion cohort and 200 mg cohort with a 28-day treatment cycle were not included in the mean plot as a 7-day dose interval was intercalated following the cycle 1 day 21 dose instead of a 2-day dose interval for the other dose cohorts.
